# Efficient Mesh Reconstruction and Texturing of Oracle Bones

**DOI:** 10.3390/s26072270

**Published:** 2026-04-07

**Authors:** Shiming De

**Affiliations:** Entertainment Technology Center, Carnegie Mellon University, Pittsburgh, PA 15213, USA; shimingd@andrew.cmu.edu

**Keywords:** oracle bones, 3D digitization, cultural heritage preservation, high-fidelity scanning, object-level reconstruction

## Abstract

The high-fidelity 3D digitization of small, detailed cultural heritage objects, such as Oracle Bones, presents significant challenges for which existing reconstruction workflows are often inadequate. Methods based on Structure-from-Motion (SfM) often lack the geometric density required to capture fine inscription details, while Light Detection and Ranging and RGB-Depth approaches may introduce high data overhead and unstable color mapping. Recent specialized studies have utilized multi-shading-based techniques to extract such hidden surface textures, yet integrating these results into a cohesive mesh remains difficult. To address these limitations, we propose a digitization framework specifically designed for object-level archaeological artifacts. Our method combines semi-automatic alignment with ICP-based refinement for robust camera pose estimation, reducing misalignment issues associated with feature-only registration. Furthermore, we employ an efficient mesh-based representation with vertex-level coloring, enabling detailed geometry and consistent texturing while maintaining compact storage requirements. Our contributions include: (1) a high-quality mesh reconstruction framework that preserves fine inscription geometry; (2) a hybrid camera pose estimation strategy that improves alignment robustness; and (3) an integrated hardware-assisted workflow tailored for digitizing small archaeological artifacts under controlled acquisition conditions. Experimental results on physical Oracle Bone artifacts demonstrate that the proposed method achieves a mean geometric reconstruction error of approximately 0.075 mm with a Hausdorff distance of 1 mm. These results demonstrate the effectiveness of the proposed workflow for digitization of oracle bone artifacts.

## 1. Introduction

The digitization of 3D cultural artifacts plays a vital role in preserving, exhibiting, and educating the public about cultural heritage. Recent advances in 3D acquisition technologies have enabled increasingly accurate digital representations of historical objects, supporting conservation and museum applications [1,2,3]. For fragile objects such as Oracle Bones, high-fidelity 3D scanning offers significant advantages because it minimizes physical handling while preserving fine geometric details. Although digitizing such object-level artifacts is crucial, most current 3D reconstruction workflows are designed for large-scale outdoor scenes, such as buildings, and fall short when applied to small, finely detailed items. While several object-level reconstruction methods have been proposed, they often fail to strike an optimal balance between reconstruction quality and processing efficiency [4]. In this context, high-fidelity 3D models provide an essential foundation for digital documentation and open science within the domain of cultural heritage [5].

Existing approaches for object-level 3D reconstruction can be broadly classified into two categories. The first relies on Structure-from-Motion (SfM), which reconstructs geometry by identifying image features, registering them across multiple views, estimating camera poses, and generating a sparse point-based representation [6,7]. Although effective in wide-baseline scenarios, such pipelines typically produce sparse or semi-dense point clouds that cannot adequately capture fine incisions and micro-reliefs—features essential to cultural artifacts like Oracle Bones. Moreover, the reconstruction accuracy heavily depends on precise camera pose estimation. Accumulated pose errors or drift often result in misalignments, which is particularly problematic for small artifacts requiring micron-level precision.

The second category involves active sensing techniques such as LiDAR and RGB-D systems, which employ time-of-flight (ToF) or structured light sensors to capture depth information [8,9]. These methods generate colored point clouds and often use Iterative Closest Point (ICP) fusion to create a unified model. While convenient, such systems present several limitations in the context of cultural heritage digitization [2,3]. First, the high data volume of colored point clouds complicates post-processing and storage. Second, because points are volumeless, a single point may be mapped inconsistently across different viewpoints, leading to unstable color assignment [10]. Finally, many LiDAR and RGB-D frameworks have been evaluated primarily on simulated or benchmark datasets [11]. Their real-world accuracy often fails to meet museum standards, revealing a significant gap between laboratory results and practical application.

In contrast to the methods described above, our approach is specifically designed to address the challenges of digitizing small, detailed cultural heritage objects [1]. Compared to SfM-based pipelines, our workflow generates significantly denser reconstructions, capturing subtle textures and fine carvings critical for Oracle Bone studies [7,12]. We combine semi-automatic alignment with fine-grained ICP refinement for camera pose estimation, effectively mitigating the misalignment issues common in feature-only registration. Relative to LiDAR/RGB-D systems, we employ a mesh-based representation that stores only vertices and indexed faces, substantially reducing storage overhead. Additionally, by optimizing color information at the vertex level, we achieve consistent and reliable texture mapping under varying viewing conditions [13]. Our main research questions are summarized as follows:

- RQ1: Can a mesh-based reconstruction framework generate high-density geometric models that preserve fine details for small object-level cultural heritage artifacts?
- RQ2: Can a hybrid camera pose estimation strategy combining semi-automatic alignment and ICP refinement improve the robustness and accuracy of registration compared with feature-only approaches?
- RQ3: Can an integrated hardware-assisted workflow provide a reliable and efficient pipeline for digitizing oracle bone artifacts under controlled acquisition conditions?

These research questions guide the methodological design and experimental evaluation presented in the following sections.

## 2. Related Work

High-fidelity 3D digitization of cultural heritage artifacts commonly relies on image-based photogrammetry, active 3D scanning, or hybrid combinations of both. Each option offers distinct trade-offs in achievable geometric resolution, color fidelity, portability, and operational robustness in museum or field settings. This section reviews representative work in Structure-from-Motion (SfM) photogrammetry, RGB-D depth sensing and reconstruction, structured-light/laser-based scanning, and hybrid pipelines, with a focus on challenges relevant to small, detail-rich artifacts (e.g., oracle bone inscriptions) [1,2,3].

### 2.1. Structure-from-Motion Photogrammetry Methods

SfM photogrammetry is widely adopted for cultural heritage documentation due to its low cost, flexible acquisition using commodity cameras, and its ability to preserve high-quality textures when imaging conditions are well controlled [7]. However, its reconstruction quality is sensitive to acquisition parameters such as image overlap, viewpoint coverage, and scene stability. Sensitivity analyses on UAV-based SfM pipelines demonstrate that insufficient overlap can significantly degrade reconstruction completeness and point-cloud quality, motivating careful capture protocols even when the target is small-scale [14]. For object-level documentation, best-practice guidelines emphasize the importance of controlled setups (e.g., turntables, coded targets/scale references, consistent lighting) to reduce drift and scale ambiguity and to improve repeatability [12]. In hazardous or constrained scenarios, SfM is also frequently combined with terrestrial laser scanning to compensate for weaknesses in either modality and to increase geometric completeness [15]. Overall, SfM remains attractive for heritage digitization, but consistently capturing sub-millimeter features on small artifacts often requires rigorous, standardized acquisition procedures [3].

### 2.2. RGB-D Sensor-Based Reconstruction Methods

RGB-D approaches integrate depth sensing (e.g., structured light or time-of-flight) with RGB imaging, enabling real-time capture and simplifying acquisition workflows. Recent learning-based reconstruction methods demonstrate that RGB-D sequences can be optimized to produce metrically consistent reconstructions with improved surface quality compared to traditional volumetric fusion, highlighting the progress of RGB-D reconstruction in controlled settings [13]. Despite these advances, RGB-D pipelines face practical challenges for high-fidelity heritage digitization, including depth noise at close range, accumulated pose drift, and texture artifacts caused by imperfect registration among frames. Robust texture mapping remains an active area of research, with methods specifically targeting misalignment-induced artifacts in RGB-D textured meshes [10]. Survey-style materials also emphasize that sensor limitations and calibration/pose quality critically shape achievable fidelity [16]. Consequently, RGB-D is often favored for rapid capture or coarse geometry, while ultra-fine details on small artifacts typically demand higher-precision active scanners or carefully controlled photogrammetry [3].

### 2.3. Structured-Light and Laser Scanning Methods

Structured-light and laser-based scanning are commonly viewed as the most reliable options for high-precision geometry capture, particularly for small objects where tiny surface relief (e.g., incised characters) is critical. Reviews of line-structured light scanning detail device- and algorithm-level considerations for achieving high precision on small-to-medium objects [8]. Comparative evaluation methodologies that benchmark photogrammetry and structured-light scanning further highlight that accuracy, completeness, and repeatability depend strongly on the scanning principle, calibration, and measurement setup [17]. In practice, deployment constraints in museums can be non-trivial: objects may be continuously displayed, access time may be limited, and lighting/space conditions can be restrictive. Methodical scanning procedures tailored for such constraints have been proposed to improve feasibility while maintaining acceptable quality under real-world exhibition conditions [9]. These observations align with broader heritage digitization discussions that stress operational planning and workflow standardization as much as algorithmic quality [1].

### 2.4. Hybrid and Multimodal Pipelines

Hybrid pipelines seek to combine complementary strengths across modalities. A common strategy is to use active scanning for accurate geometry while relying on RGB imagery for high-quality texture or to integrate SfM and laser/structured-light data for improved completeness when either modality alone suffers from occlusions or weak features [15]. More broadly, heritage digitization surveys repeatedly note that practical workflows often depend on the target’s scale, surface properties, and institutional constraints, making multimodal pipelines a pragmatic choice for maximizing fidelity under limited time and resources [2,3]. At the same time, multimodal approaches increase workflow complexity, requiring careful calibration, cross-modal registration, and additional processing stages. Nonetheless, when digitizing small artifacts where sub-millimeter features carry semantic meaning, hybrid designs remain an important direction for balancing geometric accuracy, color fidelity, and operational feasibility [1,17].

## 3. Material and Dataset Resource

**Figure 1:**
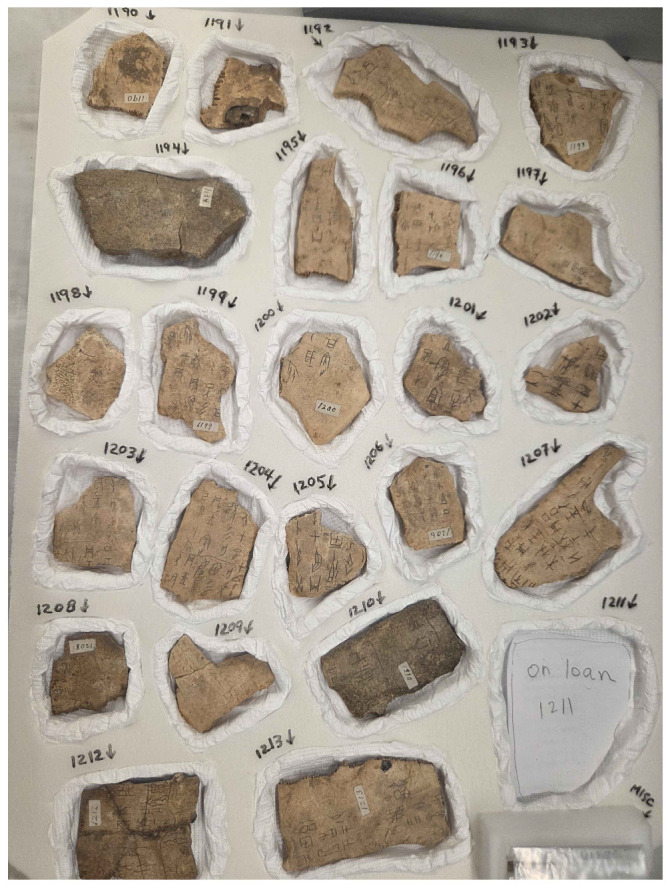
Oracle Bone Image.

The dataset utilized in this study was acquired from a collection of authentic oracle bone artifacts (see Figure 1) housed at the Carnegie Museum of Natural History (CMNH) [18]. Given their highly fragile surfaces and intricate carved inscriptions, these artifacts—including bone fragments and turtle plastrons—require critical documentation with minimal physical contact. To capture their geometric and textural information accurately and non-invasively, we employed a structured-light 3D scanning workflow. The following section details the background of the artifact collection, the hardware and software systems used, the data acquisition protocol, and the composition of the final dataset.

(1) Dataset Details. The oracle bone artifacts examined in this study are drawn from the long-term archaeological collections of the CMNH. These specimens vary considerably in size and preservation state, exhibiting characteristic inscription grooves, complex surface textures, and natural erosion marks that have accumulated over centuries. Such micro-topography necessitates a scanning methodology capable of resolving both fine-scale geometry and high-fidelity color texture. Key physical characteristics of the artifacts are summarized as follows:

- Object size: Approximately 3–12 cm in largest dimension.
- Surface color: Ranges from light yellow to dark brown, with high sensitivity to illumination and specular reflection.
- Engraving depth: Typically between 0.3 and 1.1 mm, often at a sub-millimeter scale.
- Preservation condition: Exhibits cracks, surface erosion, and fragment losses.

The combination of these properties—particularly the shallow engraving depth and reflective surface—imposes stringent requirements on the spatial resolution of the scanning system, the completeness of multi-angle coverage, and the stability of lighting conditions during data acquisition.

**Figure 2:**
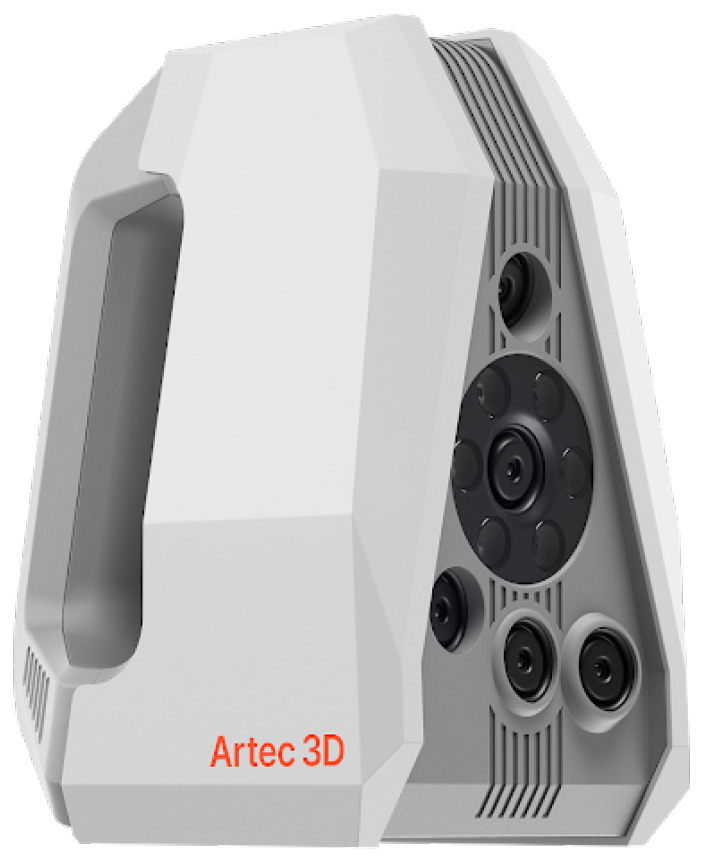
Artec Spider II.

(2) Hardware Device (see Table 1). A blue-structured-light Artec Spider II (by Artec 3D Figure 2) scanner was used to capture high-resolution geometric and texture information. Additional equipment included a motorized turntable and calibrated lighting system to ensure stable and uniform illumination.

Additional supporting devices:

- Turntable for 360° coverage
- Tripod Stand to stabilize the scanner

(3) Software Design (see Table 2). Raw scanning, preprocessing, and initial alignment were performed using the software tools listed below.

Main functional modules:

- Real-time preview of scanning coverage
- Multi-frame alignment into a unified coordinate system
- High-fidelity texture acquisition
- Noise filtering to remove isolated or reflective artifacts

(4) Data Collection Procedure. All data were collected under controlled illumination to ensure consistent color and reduce specular reflection. The data acquisition workflow includes:

1. Setup: Each artifact is placed on the turntable with calibrated lighting.
2. Multi-angle scanning: A full 360° rotation captures the top and lateral surfaces, followed by handheld scanning for occluded regions. Each artifact required 2–4 full scanning passes.
3. Frame density: merged meshes typically contain 3–12M triangles.
4. Quality verification: Coverage completeness, noise, and missing regions are inspected. Additional scans are acquired as needed.

This process ensures high-quality geometric reconstruction and minimal alignment errors.

(5) Input Description. The collected data are organized into two types of input meshes to support the semi-automated registration and fine ICP alignment stages.

(1) Original Artifact Mesh (Target)

These meshes represent the fully reconstructed geometry of each oracle bone, including:

- Complete high-resolution triangle mesh
- Detailed RGB texture
- Natural cracks, engravings, and surface erosion

(2) Preprocessed Mesh (Input)

These meshes are constructed from individual partial scans and are used as ICP inputs:

- Color-removed geometric fragments
- Uniformly downsampled point clouds
- Multiple partial views under different scanning angles

The two input sets form the basis for the subsequent semi-automated registration and fine-level optimization.

## 4. Methodology

### 4.1. Overview

**Figure 3:**
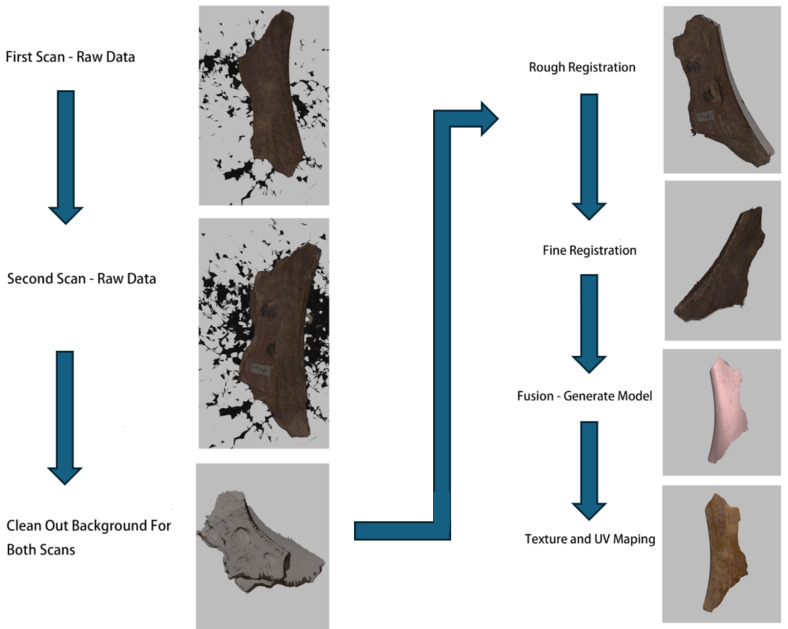
Framework of our proposed method including five main stages: Background Clean-Out, Rough Registration, Fine Registration, Fusion and Texture mapping.

The proposed five-stage pipeline (shown in Figure 3) is tailored for high-fidelity digitization of small artifacts and addresses the unique challenges of Oracle Bone scanning in museum settings. It comprises Background Clean-Out, Rough Registration, Fine Registration, Fusion, and Texture Mapping stages, which operate in sequence to produce a detailed and accurate 3D model. A mesh-based reconstruction is employed from the start, enabling the system to capture fine surface details and intricate inscriptions beyond the capabilities of standard SfM photogrammetry, while also avoiding the lower resolution and color-mapping instabilities common in commodity RGB-D scanners. The process begins with a background removal procedure that eliminates extraneous geometry (such as scanner backdrop or support surfaces), ensuring that only the artifact’s mesh remains. This is followed by a two-step alignment strategy: an initial rough registration provides coarse pose estimates for the scanned fragments, and then an ICP-based fine registration refines these alignments to achieve precise spatial correspondence. This dual registration approach overcomes the limitations of feature-based matching, yielding robust alignment even for objects with repetitive patterns or low-texture regions. Once all scans are accurately registered, they are fused into a single geometry by merging overlapping regions (e.g., by nearest-neighbor vertex correspondences), producing a unified high-density mesh representation. Finally, a texture mapping stage projects color onto the mesh, using a vertex-level coloring technique to ensure consistent color and illumination across the model’s surface. This results in a photorealistic textured model with uniform visual fidelity under varying viewing conditions—a feature particularly advantageous for museum documentation and analysis. Overall, the integrated workflow balances accuracy and efficiency, combining advanced computer-vision algorithms with practical scanning protocols to reliably digitize small cultural artifacts at high resolution.

### 4.2. Implementation Parameters

To facilitate reproducibility, the key parameters used in the registration, volumetric fusion, and texture mapping stages are summarized in Table 3. The parameter values were selected based on the scanner resolution and empirical evaluation to ensure stable alignment and accurate surface reconstruction.

### 4.3. Background Clean-Out

Raw meshes extracted from the reconstruction process often contain high-frequency noise and artifacts, manifesting as isolated floating triangle clusters disjoint from the main object. To acquire a clean geometry, we perform a topology-based filtering operation.

Specifically, we apply Connected Component Analysis (CCA) to the mesh M. The mesh is decomposed into a set of disjoint sub-meshes $\left\{S_{1},S_{2},\ldots ,S_{n}\right\}$ based on face connectivity. Assuming the target object represents the most salient geometric structure, we calculate the surface area for each component and identify the largest connected component $S_{\max}$. All other components $S_{i}$ (where $S_{i}\neq S_{\max}$) are treated as background noise and discarded. This step effectively removes outlier geometry and floating artifacts without compromising the integrity of the principal model.

### 4.4. Rough Registration Using Rigid Transformation

**Figure 4:**
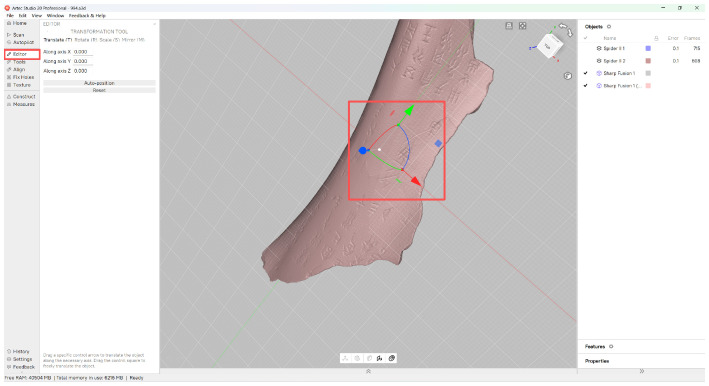
Rough registration. The highlight parts are related to the implement process.

The rough registration process involves initial coarse alignment through user interaction (Figure 4). The mathematical formulation is given by:

(1) $$M_{1}=M_{0}\cdot R+t$$

where $M_{0}$ is the original mesh model, $R\in R^{3\times 3}$ is the rotation matrix, $t\in R^{3}$ is the translation matrix, and $M_{1}$ is the final mesh model after transformation.

### 4.5. Fine Registration Using ICP Algorithm

**Figure 5:**
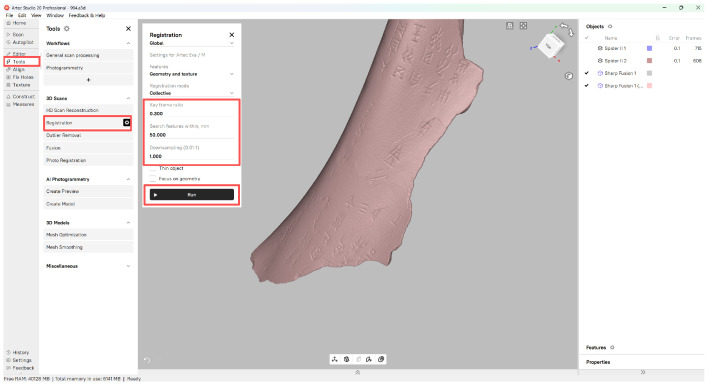
Fine registration. The highlight parts are related to the implement process.

The Iterative Closest Point (ICP) algorithm solves a nonlinear least squares (NLLS) problem for precise registration (Figure 5). The optimization objective is formulated as:

(2) $$\min_{R^{*},t^{*}}\sum_{i=1}^{N}\sum_{j=1}^{M}{∥p_{i}-\left(p_{j}\cdot R+t\right)∥}^{2}$$

where $R\in R^{3\times 3}$ is the rotation matrix, $t\in R^{3}$ is the translation matrix, *N* and *M* denote the number of points in the correspondence set $\left\{p_{i},p_{j}\right\}$, $R^{*}$ is the optimal rotation matrix, and $t^{*}$ is the optimal translation matrix.

(1) Correspondence Establishment. The first step in ICP involves finding corresponding points between the source and target point clouds. For each point in the source cloud, we identify its closest point in the target cloud using efficient nearest neighbor search methods. The correspondence can be established using various distance metrics, with Euclidean distance being the most common:

$$j^{*}=\arg\min_{j}∥p_{i}-\left(Rp_{j}+t\right)∥$$

This step is computationally intensive and often optimized using spatial data structures such as k-d trees to accelerate the search process.

(2) Translation Estimation. After establishing correspondences, we solve for the optimal translation vector. The translation component can be separated from the rotation by centering the point clouds. The optimal translation $t^{*}$ that minimizes the sum of squared distances is given by:

$$t^{*}=\frac{1}{N}\sum_{i=1}^{N}p_{i}-R\cdot \frac{1}{M}\sum_{j=1}^{M}p_{j}$$

This result shows that the optimal translation aligns the centroids of the two point clouds after rotation. The centroid of the source point cloud is computed as $\mu_{source}=\frac{1}{M}\sum_{j=1}^{M}p_{j}$, and the centroid of the target point cloud is $\mu_{target}=\frac{1}{N}\sum_{i=1}^{N}p_{i}$. Thus, $t^{*}=\mu_{target}-R\mu_{source}$.

(3) Rotation Estimation. The optimal rotation matrix $R^{*}$ is obtained by solving the orthogonal Procrustes problem. We first center both point clouds by subtracting their respective centroids:

$$p_{j}^{\prime }=p_{j}-\mu_{source},\;p_{i}^{\prime }=p_{i}-\mu_{target}$$

The rotation estimation then reduces to:

$$R^{*}=\arg\min_{R}\sum {∥p_{i}^{\prime }-Rp_{j}^{\prime }∥}^{2}$$

This problem can be efficiently solved using Singular Value Decomposition (SVD). Compute the cross-covariance matrix $H=\sum p_{j}^{\prime }p_{i}^{\prime T}$, and perform SVD: $H=U\Sigma V^{T}$. The optimal rotation is then given by:

$$R^{*}=VU^{T}$$

with the constraint that $\det(R^{*})=1$ to ensure a proper rotation. If $\det(VU^{T})=-1$, we need to correct the sign of the last column of *V* to maintain a proper rotation.

The ICP algorithm iteratively repeats these three steps until convergence, typically when the change in mean squared error between iterations falls below a predetermined threshold.

### 4.6. Triangle Fusion

After fine registration, the multiple point clouds need to be fused into a single, geometrically consistent 3D mesh. We employ a volumetric integration method based on the Truncated Signed Distance Function (TSDF), followed by surface extraction using the Marching Cubes algorithm. This pipeline ensures a watertight surface necessary for the subsequent texture mapping stage.

(1) Volumetric Representation. The physical space containing the object is discretized into a uniform 3D voxel grid. For each voxel v located at global coordinates (x,y,z), we compute its Signed Distance Function (SDF) value relative to the camera center. The SDF represents the distance from the voxel center to the nearest surface point along the line of sight.

Given the *k*-th depth map $D_{k}$ and the estimated camera pose $T_{k}=\left[R_{k}|t_{k}\right]$ from the ICP stage, the projective distance $d_{k}\left(v\right)$ is calculated as:

(3) $$d_{k}\left(v\right)=D_{k}\left(u\right)-z_{v}$$

where $u=\pi (KT_{k}^{-1}v)$ is the projection of the voxel onto the image plane, *K* is the camera intrinsic matrix, and $z_{v}$ is the depth of the voxel in the camera coordinate system. The value is then truncated to a range [−μ,μ] to focus on the surface boundary:

(4) $${\mathrm{TSDF}}_{k}\left(v\right)=\max\left(-\mu ,\min\left(\mu ,d_{k}\left(v\right)\right)\right)$$

where μ is the truncation distance.

(2) Global Fusion. To handle noise and overlapping data, TSDF values from multiple frames are fused into a global volume using a weighted running average. Let $F_{k}\left(v\right)$ be the global TSDF value and $W_{k}\left(v\right)$ be the accumulated weight after integrating *k* frames. The update rules are:

(5) $$F_{k}\left(v\right)=\frac{W_{k-1}\left(v\right)F_{k-1}\left(v\right)+w_{k}\left(v\right){\mathrm{TSDF}}_{k}\left(v\right)}{W_{k-1}\left(v\right)+w_{k}\left(v\right)}$$

(6) $$W_{k}\left(v\right)=\min\left(W_{max},W_{k-1}\left(v\right)+w_{k}\left(v\right)\right)$$

where $w_{k}\left(v\right)$ represents the confidence of the current measurement, typically proportional to the cosine of the angle between the surface normal and the viewing ray.

(3) Surface Extraction. Once the volumetric fusion is complete, the implicit surface is defined by the zero-crossing of the global TSDF field:

(7) $$S=\{v\in R^{3}|F\left(v\right)=0\}$$

The Marching Cubes algorithm is used to polygonize this isosurface. The algorithm iterates through each cubic cell in the voxel grid. The vertices of the generated triangles are positioned along the voxel edges using linear interpolation to approximate the exact zero-crossing point:

(8) $$P=P_{1}+\frac{|val_{1}|}{|val_{1}|\;+\;|val_{2}|}\left(P_{2}-P_{1}\right)$$

where $P_{1},P_{2}$ are the positions of the voxel corners on an edge, and $val_{1},val_{2}$ are their respective TSDF values.

This process transforms the discrete point cloud data into a continuous triangular mesh M=(V,F), which serves as the geometric base for the UV parameterization in the next section.

### 4.7. Texture Mapping

**Figure 6:**
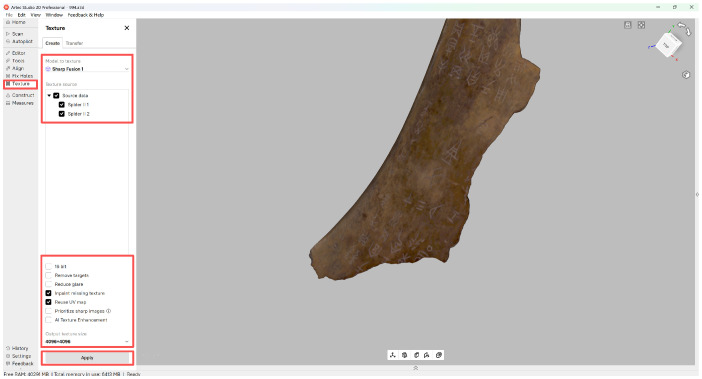
Texture mapping. The highlight parts are related to the implement process.

The process of applying UV texture mapping to a mesh model involves mapping a 2D texture space onto the 3D model surface (Figure 6). Below are the detailed mathematical formulas and steps:

(1) UV Parameterization. First, we need to establish a mapping relationship between the 3D mesh surface and the 2D texture space. Let the mesh model consist of a vertex set $V={\left\{v_{i}\in R^{3}\right\}}_{i=1}^{N}$ and a triangle face set F={(i,j,k)}.

UV parameterization can be expressed as:

(9) $$\phi :M\to \Omega \subset R^{2}$$

where *M* is the 3D mesh surface, and Ω is the 2D texture domain. For each vertex $v_{i}$, we assign a UV coordinate $u_{i}=\left(u_{i},v_{i}\right)\in {\left[0,1\right]}^{2}$.

(2) Texture Mapping Function. The texture mapping process can be expressed as a composite function:

(10) C(x)=T(ϕ(x))

where:

- x∈M is a point on the mesh surface
- ϕ(x) is the parameterization function, returning the UV coordinate of the point
- $T:\Omega \to R^{3}$ is the texture function, returning the RGB color value
- C(x) is the final color at point x.

(3) Barycentric Coordinate Interpolation. For points inside a triangle face, we use barycentric coordinates for interpolation. Let the triangle vertices be $v_{a},v_{b},v_{c}$, with corresponding UV coordinates $u_{a},u_{b},u_{c}$.

Any point x within the triangle can be expressed as:

(11) $$x=\lambda_{a}v_{a}+\lambda_{b}v_{b}+\lambda_{c}v_{c}$$

where $\lambda_{a}+\lambda_{b}+\lambda_{c}=1$, and $\lambda_{a},\lambda_{b},\lambda_{c}\geq 0$.

The corresponding UV coordinate is:

(12) $$u=\lambda_{a}u_{a}+\lambda_{b}u_{b}+\lambda_{c}u_{c}$$

(4) Texture Sampling. After obtaining the UV coordinate, we sample the color value from the texture image. Let the texture image be $I:\left[0,W\right]\times \left[0,H\right]\to R^{3}$, then:

(13) T(u)=I(u·W,v·H)

Bilinear interpolation is typically used for smooth sampling:

(14) $$\begin{array}{cc} I(x,y)= & (1-\alpha )(1-\beta )I(\lfloor x\rfloor ,\lfloor y\rfloor )+\alpha (1-\beta )I(\lceil x\rceil ,\lfloor y\rfloor )+\\ \; & \left(1-\alpha )\beta I(\lfloor x\rfloor ,\lceil y\rceil )+\alpha \beta I(\lceil x\rceil ,\lceil y\rceil \right) \end{array}$$

where α=x−⌊x⌋, β=y−⌊y⌋.

(5) Complete Texture Mapping Pipeline. Combining all the above steps, the complete texture mapping formula is:

(15) $$C\left(x\right)=I\left(\left(\lambda_{a}u_{a}+\lambda_{b}u_{b}+\lambda_{c}u_{c}\right)\cdot W,\;\left(\lambda_{a}v_{a}+\lambda_{b}v_{b}+\lambda_{c}v_{c}\right)\cdot H\right)$$

This process accurately maps 2D textures onto 3D mesh surfaces, achieving the detailed representation required for realistic rendering.

## 5. Experiments and Result

### 5.1. Main Results

**Figure 7:**
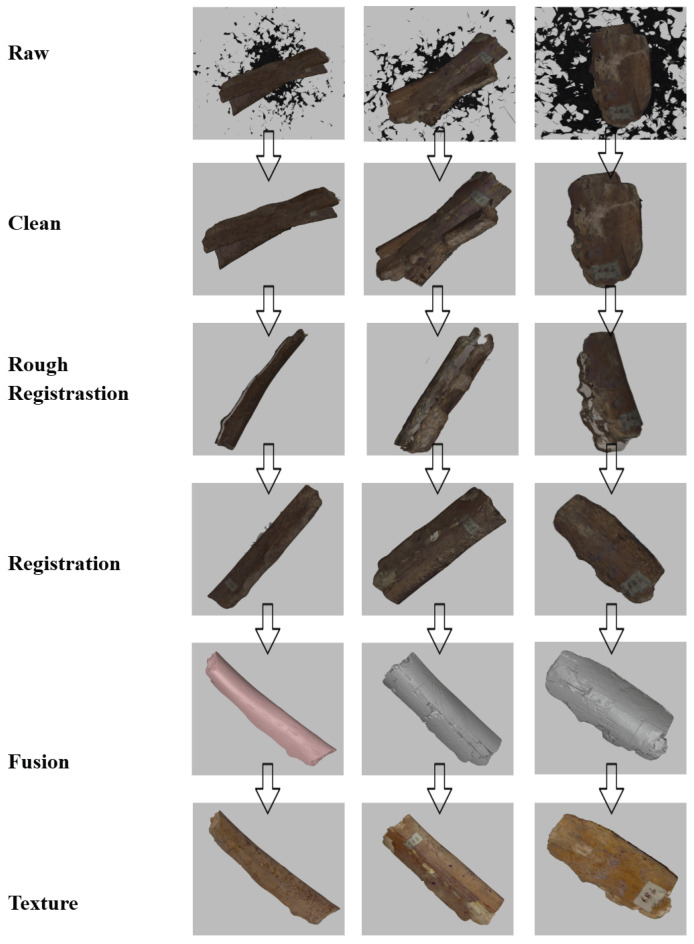
Results of three models with five mid-outputs.

Figure 7 presents the end-to-end processing results for three representative example objects (columns), with each row corresponding to a specific stage of the proposed pipeline (labeled on the left). The raw scans (top row) contain spurious background data and misaligned fragments, which are effectively removed or isolated during the *Clean* step (second row).

The *Rough Registration* stage (third row) establishes an initial alignment among multiple partial scans by introducing coarse spatial consistency, significantly narrowing the search space for subsequent optimization. Building upon this initialization, the *Registration* step (fourth row) performs fine alignment of the scans. Depending on the data condition, this stage can leverage either dense point-based matching or feature correspondence–based alignment, balancing accuracy and efficiency. In cases where the scans preserve sufficient geometric detail, exhaustive point-based registration ensures higher precision by fully utilizing available data, whereas feature-driven alignment reduces computational cost at the expense of potential residual errors introduced by correspondence uncertainty.

The *Fusion* step (fifth row) integrates the registered point clouds into a single, watertight mesh for each object. The fused geometry is visualized using a uniform color to emphasize global continuity and topological completeness. Finally, the *Texture Mapping* stage (bottom row) restores surface appearance by projecting color information onto the reconstructed mesh, producing photorealistic models that closely resemble the original oracle bone artifacts.

**Figure 8:**
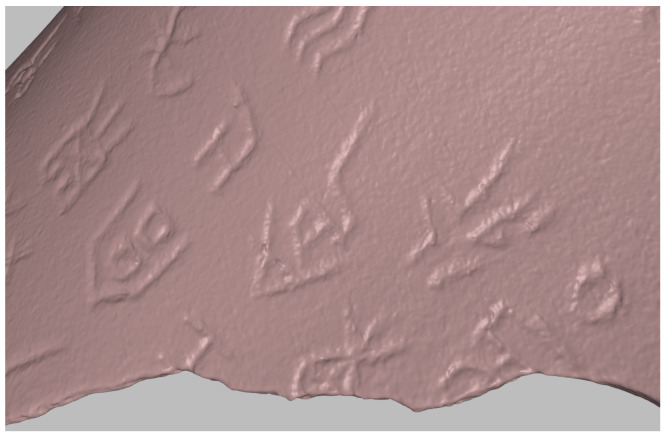
Zoom-in inscription detail without texture.

**Figure 9:**
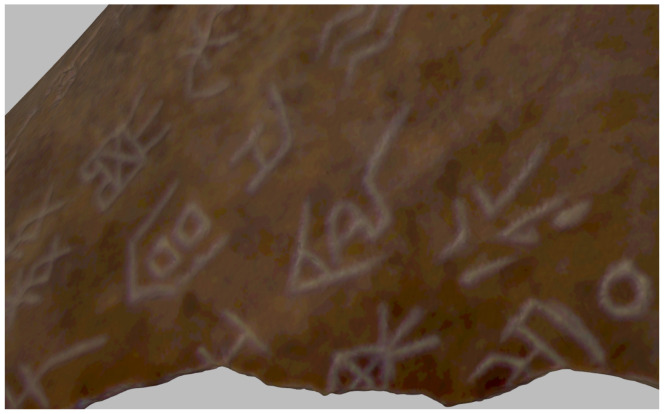
Zoom-in inscription detail with texture.

Across all three examples, the consistent improvement observed at each pipeline stage demonstrates the robustness and clarity of the proposed workflow. The final reconstructions achieve competitive quality on real-world oracle bone data, validating that the pipeline simultaneously satisfies practical requirements of efficiency, ease of operation, and high registration accuracy (Figure 8 and Figure 9).

### 5.2. Ablation

**Figure 10:**
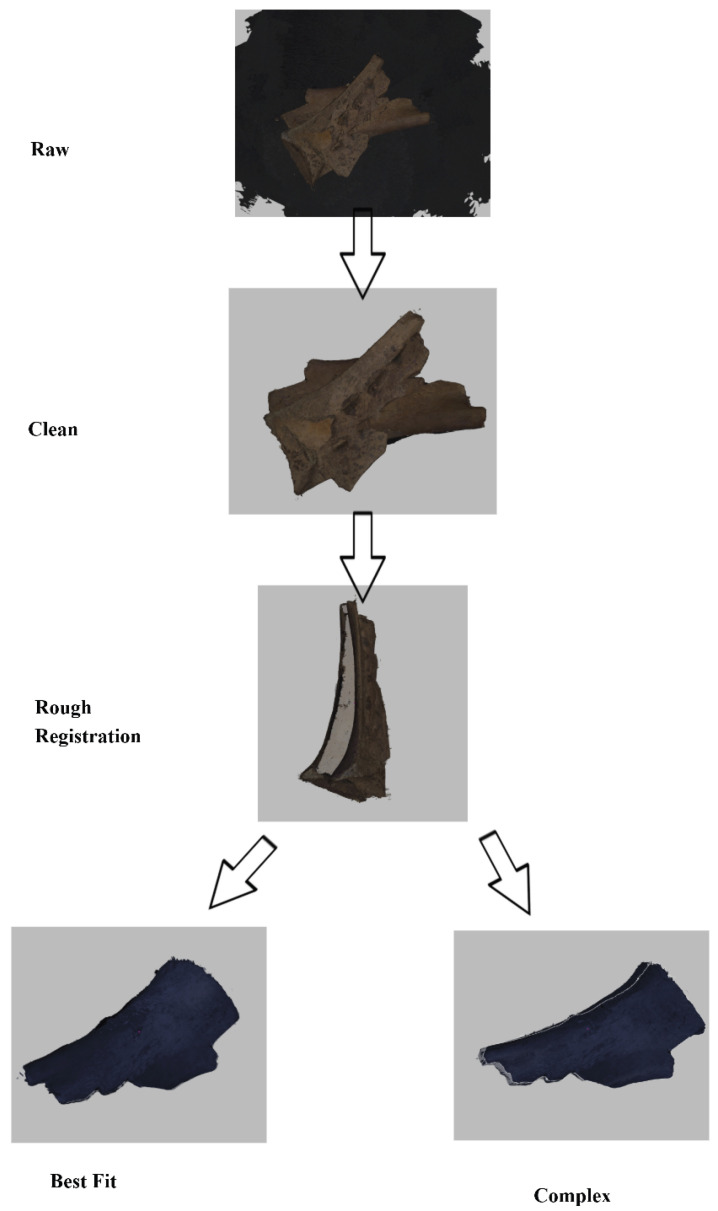
Two workflows with different registration methods including ‘best fit (ICP)’ and ‘complex (Manual Setting)’.

Figure 10 illustrates a representative object progressing from an initial raw scan to a finalized 3D model using two different registration strategies. Starting from the raw input, the scan is first denoised and trimmed to generate a cleaned model, which serves as a shared input for both alignment pipelines. From this point, two parallel registration workflows are applied.

The Best Fit (ICP) method (Figure 10, left) is a fully automatic approach that performs registration by leveraging nearest-neighbor searches over all available points. By iteratively minimizing distances across dense point sets, this method maximizes data utilization and achieves highly accurate alignment, albeit at a higher computational cost. In our oracle bone experiments, provided that the paired scans do not suffer from severe degradation, this exhaustive point-based matching consistently produces superior registration precision.

In contrast, the Complex (Manual Setting) method (Figure 10, right) reformulates the original point-to-point registration problem into a feature correspondence task. The operator manually selects a small number of corresponding feature points between overlapping scans, after which the system performs alignment based on these correspondences. This strategy substantially reduces computation time, making the Complex pipeline faster than the Best Fit approach. However, because the selected feature correspondences are subject to human uncertainty, small errors introduced at this stage can propagate through the alignment process and lead to irrecoverable residual misalignments in the final model.

Visually, both methods succeed in reconstructing complete and coherent 3D models from the same raw data. Nevertheless, the Best Fit output exhibits tighter alignment, reflected in fewer residual errors and more consistent geometric detail, while the Complex method achieves a comparable overall reconstruction in a fraction of the time. These results highlight a clear accuracy–efficiency trade-off between the two registration strategies.

### 5.3. Implement Analysis

**Figure 11:**
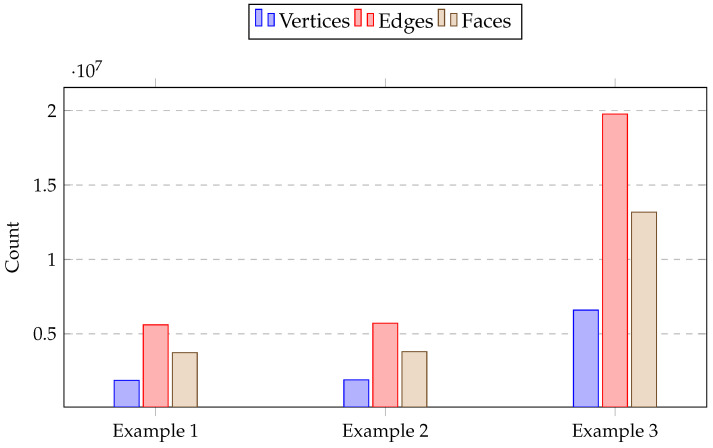
Mesh statistics for three examples.

Figure 11 presents a bar chart summarizing the number of vertices, edges, and faces for the three representative examples. As shown in the figure, the resulting meshes exhibit complete and well-defined topological structures, indicating that the reconstruction preserves full mesh connectivity rather than producing fragmented geometry. In our representation, only positional and texture-related information is stored at the vertex level, which significantly reduces the overall data size compared to representations that duplicate attributes across higher-order elements. Furthermore, texture coloring is applied at the face level, enabling consistent surface coverage across adjacent vertices. Compared to a pure point-cloud representation, this face-based texturing strategy improves robustness during rendering and downstream processing, as it maintains coherent surface information and avoids sparsity-related artifacts.

Table 4 provides a graphical summary of the execution times for key stages of the pipeline, and lists the exact timings (in seconds) for each of the three examples. Each processing stage is completed efficiently, supporting near real-time performance. In particular, the Registration phase finishes in approximately 3 s for each object, while Fusion, the most computationally intensive step, requires only 18–23 s per object. The Texture Mapping stage is also efficient, taking approximately 8–11 s. Importantly, even in the worst case, the total pipeline time per object, obtained by summing all stages, remains under 40 s. This level of performance confirms that the proposed system can generate a complete, cleaned, and textured 3D model almost immediately after data capture, making it well suited for interactive scanning applications and rapid prototyping workflows.

### 5.4. Geometric Accuracy Evaluation

To quantitatively evaluate geometric accuracy, mesh-to-mesh distance analysis was performed using CloudCompare.

The reconstructed meshes generated by the proposed pipeline were compared with the baseline meshes produced by the native Artec Studio reconstruction using the same scan data.

The reconstructed mesh was first sampled into a dense point cloud (1 M points) and compared against the reference mesh. Mean distance, RMSE, and Hausdorff distance were computed. Per-vertex error maps were visualized to illustrate local geometric deviations.

Table 5 reports the geometric accuracy results for the reconstructed oracle bone models.

The average geometric error remains below 0.01 mm, while the RMSE is approximately 0.06 mm. The maximum Hausdorff distance is mainly caused by edge regions and occluded areas during scanning.

**Figure 12:**
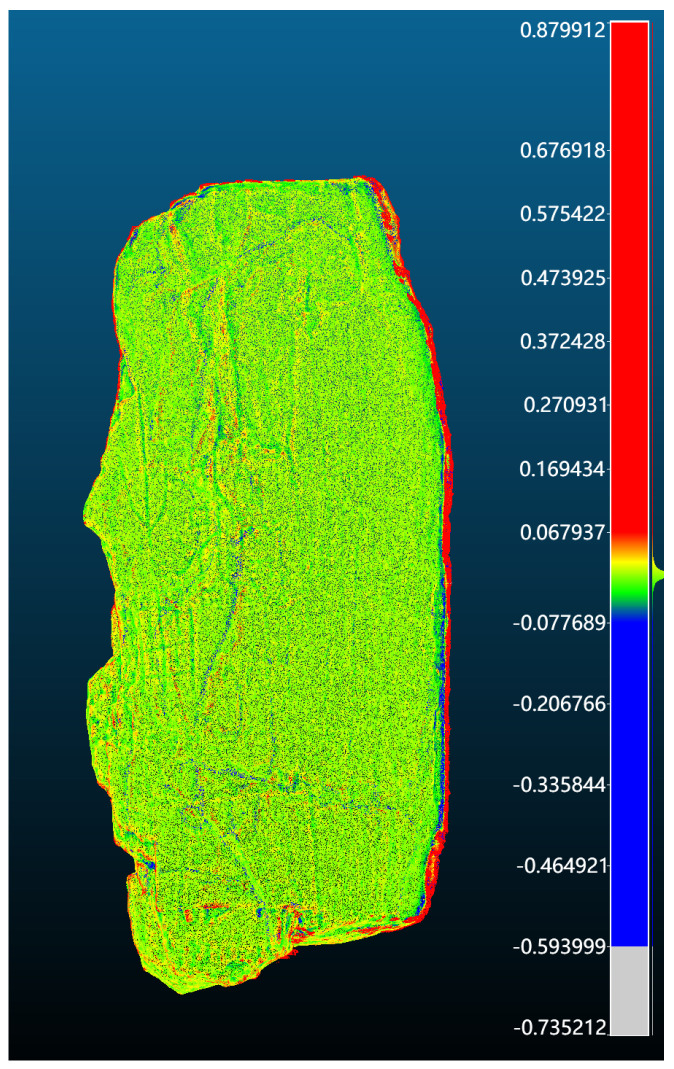
Per-vertex geometric error heatmap between the proposed reconstruction and the reference mesh produced by the Artec Studio reconstruction pipeline.

As shown in Figure 12, most regions exhibit very small geometric deviations, with larger errors appearing mainly near edges and highly curved areas of the inscriptions.

These results demonstrate that the proposed reconstruction pipeline preserves fine geometric details of oracle bone inscriptions while maintaining sub-millimeter accuracy, which is suitable for high-resolution digital heritage documentation.

### 5.5. Texture Quality Evaluation

Unlike photogrammetry pipelines, the structured-light scanner directly captures texture information together with geometry. As a result, no independent ground-truth color reference images are available for computing perceptual color difference metrics such as CIE ΔE. Therefore, color fidelity is evaluated using reprojection error and visual consistency metrics instead. Texture quality was evaluated using image-space reprojection error and visual consistency analysis. The reprojection RMSE between the reconstructed texture and the captured texture images was measured across all frames, providing an objective measure of texture alignment accuracy. To further quantify color consistency across multiple views, we measured the variance of RGB values projected onto the same surface vertices from overlapping frames. The low variance observed indicates stable color reconstruction across viewpoints.

## 6. Discussion

### 6.1. Discussion Based on Research Questions

RQ1 investigates whether the proposed mesh-based reconstruction framework can generate high-density geometric models suitable for small cultural heritage artifacts. The experimental results demonstrate that the proposed method produces dense meshes that preserve fine inscription details on oracle bone surfaces. Quantitative geometric evaluation using cloud-to-mesh distance analysis further confirms that the reconstructed models maintain high geometric accuracy relative to the reference reconstruction.

RQ2 examines the effectiveness of the hybrid camera pose estimation strategy combining semi-automatic alignment with ICP-based refinement. The results indicate that this approach improves registration stability compared with feature-only alignment methods. By incorporating ICP refinement after initial alignment, the system can effectively reduce misalignment errors and produce consistent multi-view registration results.

RQ3 evaluates whether the proposed hardware-assisted workflow can support reliable digitization of oracle bone artifacts. The experiments demonstrate that the integrated workflow provides an efficient reconstruction pipeline capable of capturing fine surface details while maintaining manageable data sizes. These results suggest that the approach is suitable for high-resolution = digitization of small archaeological artifacts under controlled acquisition conditions.

### 6.2. Limitations

Despite its successes, the current system has several limitations that temper its generality. Foremost, the framework has been developed and validated mainly on oracle bones—small, inscribed bone fragments—and its performance on other artifact types remains to be confirmed. Objects with very different material properties or surface conditions (for example, highly reflective metals or translucent glass artifacts) may pose challenges. Extremely smooth or featureless surfaces could still lead to alignment ambiguities even with ICP refinement, since the registration process relies on sufficient geometric or textural features. Additionally, the “Complex” pipeline’s reliance on a user to pick initial alignments introduces some subjectivity and requires expertise; this manual step may not scale well for batch processing large collections. Another constraint is the controlled imaging setup used in this study: each oracle bone was scanned on a motorized turntable under consistent lighting. Such conditions might be difficult to replicate for artifacts that cannot be moved from display or for in situ field digitization, potentially affecting the framework’s effectiveness outside the lab. These limitations highlight the need for careful adaptation and further testing of the system when extending it beyond the specific context of oracle bones.

### 6.3. Future Work

Building on the promising results, several avenues for future work can strengthen and expand the framework. One priority is to extend the system for higher throughput by supporting batch processing of multiple artifacts. Automating the capture and reconstruction of many pieces in sequence (or in parallel) would greatly benefit museums aiming to digitize large collections efficiently. Specifically, the high-precision geometric data generated by our framework could facilitate future research into automated Oracle bone inscription recognition and decipherment [19]. Another direction is to integrate machine learning techniques to further automate and improve the registration process [20]. For instance, deep learning-based feature detectors or matching algorithms could automatically identify key points or correspondences between scans, reducing or eliminating the need for manual alignment input. Such learned models might generalize the alignment process and enhance robustness on surfaces with weak texture or complex motifs. Finally, the framework should be adapted and evaluated on other fragile heritage materials beyond oracle bones. Applying the method to artifacts like ceramics, stone inscriptions, or delicate wooden objects will test its generalizability. These objects may introduce new challenges (e.g., different scales, reflectance properties, or surface damage) that require adjustments in scanning protocol or algorithm parameters. Addressing these issues in future research will improve the system’s versatility and confirm its value for broad cultural heritage preservation efforts.

## 7. Conclusions

In this paper, we presented an efficient 3D reconstruction and texturing framework specifically tailored for the digitization of Oracle Bones. By integrating a hybrid camera pose estimation strategy—combining semi-automatic alignment with ICP-based refinement—we successfully addressed the registration instabilities and feature-matching failures common in traditional SfM and RGB-D workflows. Our implementation of vertex-level coloring enables the generation of consistent, detailed textures while maintaining a lightweight mesh representation, effectively reducing storage overhead without sacrificing visual quality.

Experimental results on physical artifacts demonstrate that our hardware-assisted workflow provides a practical reconstruction workflow for high-resolution digitization of small oracle bone artifacts. Future work will explore the integration of neural rendering techniques and deep learning-based surface refinement to further automate the digitization process and enhance the reconstruction of increasingly complex cultural heritage objects.

## 8. Ethics and Data Availability

### 8.1. Ethics Statement

The oracle bone artifacts were scanned with permission from the Carnegie Museum of Natural History. All scanning procedures followed the museum’s handling guidelines to ensure the safety and preservation of the artifacts.

### 8.2. Data Availability

The reconstructed oracle bone meshes and derived datasets generated during this study are available from the corresponding author upon reasonable request due to museum collection restrictions.

## Figures and Tables

**Table 1:** Hardware configuration.

Title	Content
Scanner Type	Artec Spider II
CPU	Inter Core I9-13900KF
GPU	NVIDIA RTX 4090
RAM	64 GB
Hard Drive	Western Data Blue 4 TB
Interface	USB 3.0

**Table 2:** Software configuration.

Title	Content
OS	Windows 11
Scanner OS	Artec Studio 17
Output Format	.ply, .obj

**Table 3:** Key parameters used in the reconstruction pipeline.

Stage	Parameter	Value
Registration	ICP variant	Point-to-point
	Key frame ratio	0.300
	Search distance	50 mm
	Down sampling	1
	Max iterations	30
	Convergence threshold	$1\times 10^{-6}$
	Correspondence filtering	Distance-based rejection
	Sampling density	Full resolution
	Normal usage	Not used
Volumetric Fusion (TSDF)	Voxel size	0.1 mm
	Truncation distance μ	1 mm
	Weighting scheme $w_{k}\left(v\right)$	Distance-based
	Grid dimensions	3D volumetric grid
Texture	Color depth	8 bit
	Output size	4096 × 4096 px
	Reuse UV map	true

**Table 4:** Execution time (in seconds) for key pipeline stages across three examples.

	Example 1	Example 2	Example 3
Registration	3 s	3 s	3 s
Fusion	21 s	23 s	18 s
Texture	10 s	11 s	8 s
Triangles	13,178,628	3,806,344	3,737,610
Voxel Size	0.10 mm	0.10 mm	0.10 mm
Frames	1351	1575	706

**Table 5:** Geometric accuracy evaluation of reconstructed oracle bone models compared with the reference meshes generated by the Artec Studio native reconstruction pipeline.

Object	Mean Error (mm)	RMSE (mm)	Hausdorff Distance (mm)
Oracle Bone 01	0.0069	0.0597	0.8799
Oracle Bone 02	0.0238	0.1034	1.496

## Data Availability

Due to privacy restrictions, the data is unavailable for sharing.
